# Evolving models of tumor origin and progression

**DOI:** 10.1007/s13277-012-0389-0

**Published:** 2012-04-11

**Authors:** Iwona Mitrus, Ewa Bryndza, Aleksander Sochanik, Stanisław Szala

**Affiliations:** Center for Translational Research and Molecular Biology of Cancer, Maria Skłodowska-Curie Memorial Cancer Center and Institute of Oncology, Gliwice Branch, Wybrzeze Armii Krajowej 15, 44-101 Gliwice, Poland

**Keywords:** Models, Cancer, Progression

## Abstract

History of cancer disease models clearly illustrates the evolving nature of these concepts. Since such models undergo continual revisions and additions as a result of underlying medical research, they also tend to reorganize knowledge and allow perceiving previously unseen relationships. Growth of medical thought has been influenced for many centuries by an ancient Hippocratic concept of disease seen as a disturbance in bodily “humors.” True mechanisms of cell and tissue injury started to be elucidated only with the advent of postmortem pathological findings. Concerning cancer, when first disease-producing bacteria were identified in the nineteenth century, also neoplasms were treated as infectious diseases. Foreign organisms were thought to be present inside tumors. However, this hypothesis could not be confirmed by microscopic or histochemical studies. The latter suggested, instead, that tumors were rather formed by abnormal cells. Cancer was then started to be regarded as a disease of cells. This interpretation was radically altered by later developments in genetics which suggested that neoplasms can be treated as genetic diseases as pathologic cellular lesions are caused by mutations in specific genes. More recent models have compared carcinogenesis to evolutionary processes. Due to genetic instability, successive mutations, appearing in cells, lead to selection of cancer cells which feature specific phenotypic traits. The newest data indicate that there may be also a link between cancer and mutated stem cells. The review discusses main concepts of tumor origin forwarded since the beginnings of the nineteenth century.

## Introduction

Theoretical models, in general, facilitate analysis and interpretation of collected data. Such models do not describe studied processes directly, but instead, they help ordering the gained knowledge. They often suggest novel interpretations of gathered data and help perceiving unknown relationships. Owing to such models, it is easier to plan experiments and evaluate their results [1].

Cancer development and progression is a multistep process of truly great complexity. However, thanks to a continuous feedback between advances in technology and proposed cancer theories, the factual knowledge about the nature of these diseases has been continually expanding. The review focuses on the most important models of carcinogenesis advanced within the past 200 years.

## From humoral disease to the disease of organs

For long centuries, medicine has been dominated by ideas first advanced by Hippocrates and then undertaken by Galen. These ancient physicians thought that the human body was filled with four basic substances called humors, i.e., blood (sanguis), yellow bile (chole) black bile (melan chole), and phlegm (phlegm). All diseases and disabilities resulted from an excess or deficiency of one of these four substances. The root cause of cancer was seen in the surplus of black bile in the body. The humoral theory became the most commonly held view of the human body until the onset of medical research. It has been progressively eroding only since modern times [2].

Paracelsus has suggested (in sixteenth century) that responsible for the rise of tumors is the accumulation of harmful substances in the bloodstream (nowadays, this is believed to have been the first suggestion of a link between cancer and exposure to chemical agents). A century later, a new speculation, harking back to melancholism, was put forward. It held that tumor development is caused by coagulation and fermentation of blood or lymph. With time, possible causes of cancer begun, including chronic inflammation and injuries as well as familial predispositions and traumas [3].

The development of true medical knowledge was strongly influenced by pathologists performing postmortems. Among the first men attaching a right measure of importance to these findings was a famous Italian anatomist, Giovanni Battista Morgagni. In 1761, he published a famous treatise (*De Sedibus* *et Causis* *Morborum per* *Anatomem Indagatis*) which characterized the course of various diseases and linked their symptoms with subsequent autopsy findings [2]. Morgagni believed that causes of a disease (not only cancer) involved pathological lesions of a particular organ. Autopsies thus contributed to the abandonment of humoral theory in favor of perceiving disease as an “organ lesion.” Gross pathology evolved rapidly in the nineteenth century, owing largely to the works of an eminent Vienna-based physician Rokitansky who is said to have performed some 20,000 autopsies in his lifetime [4].

Fast advances in gross pathology were matched by those in microscopic pathology as the nineteenth century saw a more widespread use of microscopes. First, such instruments were constructed toward the end of sixteenth century. Some 50 years later, Hook described, using one of them, structures found in biological material and termed them “cells.” It is van Leeuwenhoek (seventeenth century), however, who is thought to have been the father of microscopy and who described microorganisms which he called “animalcules” [5]. Manufacture of an achromatic lens, in 1830, allowed constructing microscopes with greater magnifying power [6], which soon led to great advances in bacteriology and cellular-level pathology.

## Cancer as a parasitic disease

Until nineteenth century, no one realized the importance of microorganisms in the disease process. Halfway into that century, though, thanks to the works of Pasteur and Koch, the relationship between bacterial infection and disease was recognized [7].

Discovery of disease-causing bacteria has started a quest for cancer “germs” in the late nineteenth century. It was believed that the growth of tumors might be caused by foreign organisms (bacteria, fungi, or roundworms) living within such tumors. Toxins released by them were supposed to emaciate the host. Metastasis was explained as parasites attacking consecutive organs. Observed increased cancer frequency among some families was explained as the effect of more frequent contact (either cross infection or exposure to the same parasite) between related individuals [8].

Attempts to isolate cancer germs were undertaken numerous times. The best-known (and honored by Nobel Prize) attempts were described by Fibiger. He studied stomach tumors in rats fed with cockroaches infected by *Spiroptera neoplastica* worm. The origin of these artifacts remains unknown. It is likely that Fibiger mistook tumors with other parasite-caused pathological lesions [9].

## Cancer as a disease of cells

Techniques of preparing histological specimens (frozen and fixed sections), as well as staining of biological material, were introduced in the mid-nineteenth century. Fixing tissues with paraffin was invented by Klebs in 1869, whereas formalin was introduced into laboratory practice by Blum in 1894. Besides, dyes like carmine, extracted from cochineal and already used by van Leeuwenhoek, new ones were added (picric acid, basic fuchsin, eosin, and hematoxylin). Double-staining methods (hematoxylin and eosin (H&E)) were introduced [10, 11]. These dyes not only permitted intense development of bacteriology, but they also allowed a more precise description of tissue and cellular structures [4].

Structure of tumors was described in greater detail. In the first half of the nineteenth century, several researchers observed that neoplastic tumors are made up of cells with abnormal morphology. Such findings were reported by Schleiden and Müller (in 1838) and Schwann (in 1839) [12]. Excessive number of dividing cells, anomalous mitoses, oversized cell nuclei, and loss of differentiation were all listed as characteristic features of neoplastic tumors. Other features, such as tumor stroma, vasculature, and necrosis seen in central part of tumors, were also noticed [8]. Toward late nineteenth century, more and more researchers gravitated toward the theory proposed by R. Virchow, i.e., that the underlying cause of the disease lies in cellular lesions.

Thus, cancer started to be treated as a disorder of abnormally dividing cells. They were thought to form aggregates that mechanically damaged neighboring tissues. Furthermore, cancer cells used up nutrients and poisoned the organism with toxins [8]. Because of this, some researchers were considering cancer cells as a peculiar kind of “parasite.”

Appearance of metastases was seen as a result of detachment of cancer cells from tumor mass. Such cells circulated in blood vasculature until they became trapped in distant sites. The role of lymphatic vessels in metastasis was described by Brouns in 1847 [13]. A theory of “seed and soil,” credited by some to Paget, was put forward in 1889. According to this theory, cells (“seed”) detached from tumor mass must find a suitable place (“soil”) in the body in order to continue growth. The necessity of finding appropriate soil would explain why particular types of cancer metastasize into specific locations [13, 14].

The origin of neoplastic cells was unknown. Cohenheim postulated that these cells could be actually embryonic cells that were not eliminated during ontogenesis. Hanseman believed that they could be cells that underwent dedifferentiation (anaplasia). It was even posited that cancer cells could originate from a different organism; they somehow managed to penetrate the body of a diseased individual (Kelling). Irrespective of origin issue, it was conceded that uncontrolled divisions began as a result of external physical and chemical stimuli (burn, X-rays, or certain chemicals) [8, 12].

## Cancer as a disease of genes

Treating cancer as a disease of cells brought up the question: what is the cause of abnormal proliferation of cancer cells? Inappropriate number of chromosomes or their incorrect structure was often observed when studying cancer cells. Examination of chromosomes started in the mid-nineteenth century, even before their role in heredity was established. At the beginning of twentieth century, Boveri hypothesized that carcinogenesis is triggered by chromosomal aberrations in cells. In 1910, Johannsen proposed the term “gene” instead of “hereditary factor.” It was only in 1926 that Muller demonstrated the mutagenic effect of X-rays, although de Vries suggested the existence of mutations already in 1901. In the 1940s of the last century, chromosomal DNA, not protein, was shown to be the carrier of genetic information. In 1953, Watson and Crick [15] described the structure of DNA. Within the last 50 years or so, semiconservative character of DNA replication was proven [16], genetic code was learned (Holley, Khorana, Nirenberg) [17], and finally, in the 1970s, DNA cloning and sequencing techniques emerged.

Development of genetics had immense impact on the understanding of cancer causes. In the 1960s of the last century, it was learned that cancer development is linked to errors contained in the genetic material and that these errors are transferred to progeny cells during subsequent divisions [18]. Thus, interpretation of carcinogenesis shifted from cellular (disease of cells) to molecular level (disease of genes).

At the turn of the 1970s and 1980s, a new model of cancer disease was proposed. Accordingly, the disease started from a single cell in which a mutation occurred. Such cell divided under the influence of proliferation-stimulating factors (e.g., growth factors, hormones), forming a population of similar cells (clone). Tumor progression was caused by subsequent mutations occurring in cells, forming the clone. Clones differing from each other appeared. Some clones might undergo growth factor-independent proliferation and this triggered rapid acceleration of tumor growth. Since no key carcinogenic genes were known, the proposed model could only describe phenotypic cell alterations and not specific mutations [19].

Search for factors increasing the risk of cancer development has been ongoing since the nineteenth century. As far back as in mid-eighteenth century, Hill noticed increased frequency of cancer in smokers. In turn, Pott described frequent occurrence of certain type of cancer in chimney sweepers, which he explained to be the result of their exposure to harmful substances present in sooth (including sulfur and arsenic) [20]. With time elapsed, other agents were added to this list (certain chemicals, e.g., pitch and X-rays) [8].

The mechanism of carcinogenic action has long remained totally unknown. Explaining it became possible only with the advent of knowledge about DNA structure. In the late 1960s of the twentieth century, it was demonstrated that certain carcinogens have the ability to bind DNA. Genotoxic compounds damage DNA (e.g., by forming adducts or causing DNA strand breaks). It has been known now that certain carcinogens do not act on DNA directly; instead, they induce free radicals or activate signaling pathways leading to increased proliferation or inhibition of apoptosis [20].

A separate category of carcinogens is that formed by viruses. In 1907, Ciuffo demonstrated that an infectious agent responsible for human warts is transferred by cell-free filtrates. He could not identify the pathogen, though. At the beginning of twentieth century, it was noticed that chicken leukemia is infectious. However, the attempts to isolate the responsible pathogen proved futile (it was actually avian leukemia virus), and leukemia was not treated as cancer at that time. In 1911, Rous conducted some experiments during which he injected chickens with cell-free sarcoma extract which led to the development of cancer. Rous thus became the discoverer of the first oncogenic virus; it was termed Rous sarcoma virus (RSV). In the 1930s of the previous century, subsequent animal cancer-causing viruses were discovered. In the early 1960s, relationship was demonstrated between Epstein–Barr virus infection and Burkitt lymphoma in humans. The earlier mentioned observation made by Ciuffo was explained in the 1970s; the responsible pathogen turned out to be human papilloma virus. Similar correlations were later found for other viruses and kinds of cancer (for example, hepatitis B increases the risk of hepatocellular carcinoma, and HHV-8, the risk of Kaposi's sarcoma) [21, 22].

Research performed in the 1960s demonstrated a link between viral infection and acquisition of cancer cell-like features by infected cells. In vitro experiments had shown that expression of viral proteins in normal cells led to their accelerated proliferation and ability to undergo unlimited divisions. Cells infected with oncogenic viruses developed numerous chromosomal aberrations [23]. First viral oncogenes were discovered (among them *SRC* from RSV) [22].

Presence of viruses increases the risk of acquiring cancer, but is not its direct cause. Incorporation of viral genetic material into the host genome may lead to oncogenic transformation. Presence of viral proteins changes the phenotype of host cell. Another mechanism is also possible; integration of retroviral DNA into genomic DNA can lead to changes in protein expression. The resulting excess or lack of some proteins can also be the cause of pathologic lesions [21].

Mutations leading to carcinogenesis may be caused, for example, by replication errors during cell division or differentiation [19]. The model of treating cancer as a disease of genes has allowed explaining the link between exposure to mutagenic agents and morbidity and provided explanation for increased frequency of some cancers in certain families as well as for increased incidence among the elderly.

Initially, it was not known which genes play key roles in carcinogenesis. Researchers supposed that responsible genes encoded proteins involved in DNA damage repair, in proliferation, and perhaps genes encoding surface receptors, hormones, etc. To study the role of particular genes in cancerogenesis, genetically modified mice with inhibited or overexpressed genes were used. In some experiments, animals were infected with viruses carrying specific genes or administered with virally modified cells. If cancer developed, it was thought that the examined gene played some role in carcinogenesis. Additional detailed experiments verified the role of such a gene [24].

The 1980s were a period of intense studies on genes involved in carcinogenesis. It was noticed that several genes found in oncoviruses and the counterpart cellular genes possess identical sequences (e.g., *SRC*, discovered in avian DNA) [25]. This meant that viruses can incorporate in their genome proliferation-stimulating genes of cellular origin [22, 26].

Structure and function of such genes as *TP53*, *C*-*MYC*, and *C*-*RAS* were discovered. It was found that RAS and MYC proteins stimulate cell proliferation, whereas TP53 is involved, among other things, in the control of cellular response to DNA damage (halting cell division and committing affected cells to apoptotic pathway). Genes involved in carcinogenesis were classified as suppressor genes (antioncogenes) and protooncogenes. The latter encode proteins that stimulate cell divisions. If mutation occurs, these proteins can be expressed continuously, leading to uncontrolled proliferation. The products of suppressor gene expression, in turn, inhibit cell division and survival [27].

It is currently thought that the initiation of carcinogenesis requires simultaneous activation of protooncogenes and inactivation of suppressor genes. An important role in oncogenesis is ascribed to mutations in genes encoding proteins taking part in apoptosis, detection, and repair of damaged DNA. It has been assumed that cancer results from several mutations (4–7) in such genes; accumulation of mutations seems to have priority over the order in which they appear [28].

## Carcinogenesis as evolutionary process

Hanahan and Weinberg listed several phenotypic features and complex processes, distinguishing cancer cells from normal ones. Among them are: self-sufficiency in terms of growth signal dependence, insensitivity to growth inhibitors, resistance to apoptosis, limitless replicative potential, sustained angiogenesis, and ability to invade neighboring tissues and form metastases [29]. The list of phenotypic properties of cancer cells has been expanded. Cancer cells can survive under stress conditions, like hypoxia or nutrient deprivation. They tolerate DNA damage and presence of incorrectly folded proteins. Errors during mitosis, such as incorrect separation of chromosomes between daughter cells, frequently do not result in apoptosis commitment [30]. Cancer cells can escape from immune surveillance. Besides, they are able to transform their microenvironment in a way that favors tumor progression [31]. All these features are collectively termed “hallmarks of cancer.”

It was noticed in the 1970s of the twentieth century that cells forming tumor mass are very heterogenous. They differ, for example, in their resistance to drugs or sensitivity to proapoptotic signals. In addition, they become, in time, more and more aggressive and acquire new features, such as an ability to form metastases. The model of cancer disease as a “disease of genes” has taken into account the role of mutation in disease initiation, but never fully explained this process. It was concluded that there must be a mechanism facilitating the emergence and accumulation of successive alterations. In 1976, Nowell compared neoplastic progression to evolutionary process [32].

Cancer cells undergo clonal selection. Since starting cells contain various mutations, also their clones differ between them. Majority of genome alterations are phenotypically either neutral or lethal; only few of them favor cell survival. Such clones start to dominate the growing tumor. Features giving cells a selection advantage accumulate during tumor progression. One of such features appearing in cancer cells is genome instability [33]. Owing to this feature, risk of successive mutations is increased. Clonal evolution leads to emergence, from the population of cancer cells, of clones best adapted to conditions found within a tumor mass [34].

One of the primary factors favoring cell selection is oxygen and nutrients' deprivation. In the initial stage of tumor growth, cancer cells form small avascular aggregations of cancer, 0.5–1 mm in diameter, the so-called in situ tumors [35]. Only cells using anaerobic glycolysis as main energy source are resistant to lack of oxygen and acidic environment and can survive at this stage. Because of anaerobic glycolysis, the demand for glucose becomes increased, whereas that for oxygen decreases (Warburg effect) [36]. Such cells tend to accumulate lesions that favor survival under unfavorable conditions. Phenotypic changes can result from the appearance of new mutations or they can be due to altered gene expression or interference with signaling pathways [37].

This picture is radically altered when tumors suddenly acquire the ability to form their own blood vessels (angiogenic switch). This can be caused by increased amounts of proangiogenic factors (such as VEGF) secreted by cells or by decreased production of antiangiogenic molecules. Formation of tumor vasculature is favored by the presence of other proteins such as metalloproteinases, degrading extracellular matrix and facilitating migration of endothelial cells. Owing to a newly formed blood vessel network, tumor cells are supplied with nutrients. This leads to a quick increase of tumor mass which includes neoplastic cells with proangiogenic phenotype [35].

Functioning of the immune system is an important selection factor in cancer. Initially, selection favors abnormal cells avoiding immune recognition and destruction, i.e., cells displaying low expression of cancer-related antigens. At later stages, cancer cells not only avoid immune recognition, but they also produce cytokines and chemokines via which they carry a specific “talk” with cells forming tumor milieu [38]. Influx of various cells, such as monocytes, macrophages, dendritic cells, and fibroblasts, takes place in tumors. Under the influence of cytokines and other factors secreted by these incoming cells, the residing neoplastic cells can acquire novel properties. Also, the incoming cells can alter their phenotype. Tumor-associated macrophages (TAMs) start producing proangiogenic and immunosuppressive factors such as VEGF, TGF-β, or IL-10. Other type of affected cells includes fibroblasts. These cancer-associated fibroblasts (CAFs) also start producing proangiogenic and immunosuppressive cytokines and participate in the reconstruction of extracellular matrix within tumors. The emerging tumor milieu facilitates tumor progression [39].

## Cancer as a disease of stem cells

One of the most recent models of tumorigenesis assumes that the origin of cancer lies in excessively proliferating and abnormally altered stem cell. In the 1960s of the last century, existence of organ-specific stem cells was demonstrated. A hierarchy of cancer cells was described 10 years later; only part of tumor cells is capable of infinite growth, while the majority has a limited replication potential [40].

Organ-specific stem cells, by assuring tissue renewal, enable functioning of the whole organism. These cells possess two defining properties: capability of self-renewal and ability to differentiate into other cells [41]. Despite a very efficient system of DNA damage repair, these cells can also accumulate mutations. Damage to suppressor genes and protooncogenes can initiate tumorigenic process [40]. Proliferation and differentiation of stem cells are significantly affected by tumor niche. Changes in tumor microenvironment (e.g., disturbed signaling pathways) can also trigger excessive proliferation of stem cells [42].

One of the basic features of cancer stem cells (CSCs) is their ability to form clones in vitro as well as experimental tumors in vivo (in such trials, human cells are injected into immunodeficient mice). Cells showing the discussed features were found in numerous kinds of tumors (lung, prostate, breast, and others) [43]. However, the existence of CSCs is not a proof that cancers originate from them. Another path leading to the emergence of cells with the above-described features can involve clonal selection; as a result of accumulated mutations, part of the differentiated cancer cells acquire the ability to divide continuously and to further differentiate, i.e., features characterizing stem cells [41]. CSCs can emerge as well as a result of dedifferentiation. Epithelial-mesenchymal transition (EMT) provides a mechanism leading to the appearance of cell motility and certain features specific for stem cells. Such transition occurs under the influence of different agents (including hypoxia) which may be present in various areas of neoplastic tumors [44].

## Cancer as a systemic disease

Presence of a tumor affects the functioning of entire body; some researchers believe that the causes of cancer, as well as its particular phases, require taking a wider look than just that focusing on processes affecting tumor itself. Even at the time when cancer was regarded as a disease of cells, many judged this model as oversimplified. The body is not just a “sum” of its component cells. The direct causes of cancer might lie in disturbances of homeostasis: cancer is no more a disease of cells that a traffic jam is a disease of cars [45, 46]. One of the arguments supporting this concept concerns cases of spontaneous healing. According to cell theory, they should not occur at all; an organism is capable, however, to regain equilibrium (homeostasis) in some cases. It has also been known that some cancers (notably breast and prostate tumors) are hormone-dependent, and therefore, hormonal disorders can be treated as homeostatic disturbances [46]. It is not clear, however, what is the direct cause prompting the development of cancer. Since increased cancer incidence has been noted among people with impaired immune system (e.g., taking immunosuppressants or HIV infected), perhaps, this makes immune cells unable to recognize and destroy cells carrying viral genetic material [47].

Tumors contain, besides abnormal cells, extracellular matrix that differs from that present in normal tissue, both in terms of structure and protein composition. Its volume vs. that of cells is greater than in normal tissues [48]. This results in altered adhesion of neoplastic cells, expression of numerous proteins, and signaling pathways. Tumor could thus be regarded as emanation of incorrect tissue growth [49, 50].

It has also been pointed out that tumors bear similarities to unhealing wounds. As during wound healing, tumor cells are exposed to hypoxia; neovascularization takes place along with remodeling of extracellular matrix and influx of immune system cells. As during inflammation, tumor blood vessels are leakier than those in normal tissues, and serum proteins infiltrate surrounding tissues. Interstitial pressure in tumors becomes elevated. Obviously, these processes occur in tumors in a pathological manner [51].

## Summary

Models discussed in the paper illustrate how cancer understanding has evolved in time and how various discoveries have contributed to this evolution. Subsequent models have reflected medical knowledge attained at corresponding time points. For example, acquired knowledge of bacteria role in infectious diseases triggered a search for cancer causes among microorganisms. This cancer model was later abandoned, owing to advances in pathology at cellular level. Our perspective on cancer causes was drastically altered with the elucidation of DNA structure and role. This discovery helped to explain observations previously seen only as a cause–effect relationship (e.g., the role of viruses and carcinogens in pathogenesis). This cancer concept was subsequently reinforced by incorporating in it the idea of clonal selection which explains well the propensity of cancer cells to acquire successive mutations. Current cancer models attempt to incorporate the most recent knowledge about stem cells and microenvironment of immune system cells during cancer progression. Hopefully, future cancer models will step in the wake of technological breakthroughs and describe in ever greater detail the most crucial events taking place in cancer cells as well as whole tumors. In any case, diagnostics and therapy seem to be clear beneficiaries of future developments in this area.
